# The time-course changes of NT-proBNP and tissue Doppler indices in patients undergoing mitral valve replacement

**DOI:** 10.5830/CVJA-2011-057

**Published:** 2012-05

**Authors:** DR Prakaschandra, DP Naidoo, T Esterhuizen

**Affiliations:** Department of Biomedical and Clinical Technology, Durban; University of Technology; and Department of Cardiology, University of KwaZulu-Natal, Durban, South Africa; Department of Cardiology, University of KwaZulu-Natal, Durban, South Africa; Department of Biostatistics, University of KwaZulu-Natal, Durban, South Africa

**Keywords:** mitral regurgitation, tissue Doppler imaging, NT-proBNP, mitral valve replacement

## Abstract

**Background:**

In severe mitral regurgitation, a subset of patients who are asymptomatic may develop left ventricular decompensation before changes in echocardiographic parameters become evident. Since N-terminal brain natriuretic peptide (NT-proBNP) is used to detect early heart failure, we hypothesised that NT-proBNP would be activated in patients with mitral regurgitation.

**Methods:**

Patients submitted to surgery were prospectively evaluated over eight months in the Department of Cardiology at Inkosi Albert Luthuli Central Hospital. Control patients with severe mitral regurgitation were obtained from the outpatient clinic. In order to define their value in identifying left ventricular decompensation, NT-proBNP levels and tissue Doppler imaging (TDI) indices were simultaneously measured and compared with conventional echocardiographic indices at baseline and this was repeated at one week and at six weeks after valve replacement.

**Results:**

Mean NT-proBNP levels were markedly elevated pre-operatively in all surgical cases compared to controls (*p* = 0.0001). The diastolic E-mitral/E-annulus ratio, measured using TDI, was higher in the study group, indicating higher left ventricular filling pressure present in the study group. NT-proBNP levels increased further at one week after surgery and subsided at the six-week follow-up visit to levels similar to the control group. The TDI diastolic ratio also decreased at one week, and increased slightly again at the six-week follow up. These changes were accompanied by significant reduction in left atrium and left ventricular chamber dimensions with an increase in the ejection fraction from one to six weeks.

**Conclusion:**

Marked differences in mean NT-proBNP levels and TDI ratios between the study and control groups suggest that using TDI and NT-proBNP assays may detect covert left ventricular decompensation.

## Abstract

Chronic organic mitral regurgitation (MR) has a variable course and requires careful monitoring by the clinician. Symptoms often occur late due to the compliance properties of the left atrium that allow it to accommodate large volumes of blood without a significant rise in pressure. As the regurgitation becomes more severe, contractile dysfunction may precede the onset of symptoms associated with disease progression as the ejection fraction (EF) declines but it may still remain in the normal range. An EF less than 60% has been shown to be associated with poorer survival rates after corrective surgery and is likely to indicate covert contractile dysfunction in MR patients.^1^

Although numerous qualitative and quantitative echocardiographic modes have been developed, previous studies have demonstrated that existing measures of severity of MR correlate poorly with clinical signs and symptoms.^2^ There are few data on the newer echocardiographic modalities, notably tissue Doppler imaging (TDI), the predictive values of which have not been determined. While the mitral inflow measurements are preload-dependent, diastolic tissue velocities measured using TDI are far less influenced by these parameters and may be more reliable in assessing contractile changes.

The development of contractile dysfunction and its relation to the severity of volume overload in MR is not clearly understood. Prolonged contractile dysfunction eventually becomes irreversible even after the MR is corrected and is predictive of both congestive heart failure and death.^1^ In chronic organic MR, there are as yet no clear definitions of BNP physiological determinants and outcome implications. We hypothesised that N-terminal brain natriuretic peptide (NT-proBNP) would be activated in MR and, because this is a validated diagnostic test in heart failure, it could prove to be an early marker for the development of left ventricular (LV) dysfunction.

In this study we evaluated tissue Doppler imaging and NT-proBNP levels in patients with severe chronic MR and determined their time-course patterns following mitral valve replacement.

## Methods

The study population was selected from Inkosi Albert Luthuli Central Hospital, Durban. Informed consent was obtained from each patient, and in the case of minors, from the parent or legal guardian. The study protocol conforms to the ethical guidelines of the 1975 Declaration of Helsinki. Ethics approval was given by the Biomedical Research Ethics Committee at the University of KwaZulu-Natal, Nelson R Mandela School of Medicine (Ref No. H112/06).

The study enrolled patients prospectively from February to September 2007. Patients with severe, chronic isolated MR underwent comprehensive quantitative Doppler echocardiography performed by one trained echocardiographer (RP). Patients were excluded if they had acute MR, MR due to ischaemic heart disease or cardiomyopathy, previous valve surgery and associated aortic or congenital valve disease. Patients with associated mitral stenosis were excluded if the valve area was less than 2.0 cm^2^.

Clinical evaluation and management of the patients were conducted by their independent clinicians. Assessment of symptoms was determined clinically by the New York Heart Association (NYHA) classification, and atrial fibrillation was evaluated by electrocardiography. Doppler echocardiographic recordings and blood samples were collected simultaneously and estimation of the NT-proBNP levels were processed independently.

Control subjects with severe MR were selected from the cardiology outpatient follow-up clinic where they were assessed as not requiring surgery in the short term and were receiving medical therapy. Controls were selected as severe MR, but without evidence of ischaemic heart disease, as it is known that NT-proBNP is activated in the presence of ischaemia.^3^

Colour Doppler echocardiography was performed on all patients using a Siemens Sequoia machine (Acuson, Germany). Dimensions and wall thickness were measured according to the American Society of Echocardiography guidelines using the leading-edge method,^4^ and EF was measured from the apical four-chamber view using the modified Simpson’s method. The rate of rise of pressure (dP/dT) was also calculated as a measure of ventricular systolic function. The six frequently applied echocardiographic variables described by Thomas *et al*.^5^ were used to evaluate MR. Mitral regurgitation was quantified by measurement of the regurgitant volume and fraction.

TDI was performed in the apical four-chamber view. The myocardial systolic wave (Sm) velocity, the diastolic indices, namely early myocardial (Em) and atrial contraction (Am) peak velocities, Em/Am ratio, and early diastolic filling ratios (transmitral/annular) (Em/Ea ratio) were measured. The ratio of the E wave across the mitral valve to the annulus E wave on tissue Doppler (E/Ea) was used as an estimate of the LV filling pressure. All echocardiographic measurements were performed by a single echocardiographer (RP) blinded to all other measurements. The images and measurements were reviewed off-line by a trained cardiologist (DPN). The intra-observer variability for the measurements of proximal isovelocity surface area (PISA) and TDI was < 5%.

For measurement of NT-proBNP, venous blood samples were taken in gel-filled tubes with the patient resting quietly at the time of echocardiography. Additional samples were taken from subjects undergoing mitral valve replacement at one and six weeks post surgery.

## Statistical analysis

SPSS for Windows version 15.0 was used for the statistical analysis. Clinical variables were normally distributed and expressed as mean ± SD. NT-proBNP levels were log-transformed for statistical analysis. Group comparisons were performed with ANOVA, *t*-test or chi-square tests. Associations of baseline NT-proBNP were tested with linear and non-parametric regression (categorical variables). The time course of NT-proBNP within the cases was evaluated using paired *t*-tests. The discriminating capacity of the NT-proBNP for separating surgical cases from controls was assessed by the construction of ROC curves. Bivariate analysis was performed to assess the ability of the different parameters in predicting a favourable outcome using an NT-proBNP level of 50 pmol/l.

## Results

A total of 54 patients with severe rheumatic mitral regurgitation were enrolled in the study and surgical cases were followed up for six weeks. Their baseline characteristics are shown in Table 1. All but one of the patients in our sample population were of African descent (98%). There were 27 control patients with severe chronic rheumatic mitral regurgitation who were recruited from the cardiology follow-up clinic.

**Table 1:** Baseline (Pre-Operative) Characteristics In Severe MR

*Variables*	*Controls (n = 27)*	*Study (n = 27)*	*p-value*
Age	23 ± 13	20 ± 11	0.08
Males/females	7/20	7/20	1.000
NYHA I–II	24	10	0.001
NYHA III–IV	3	17	
Heart failure	1	5	
EF (mean ± SD)	67 ± 6	67 ± 9	1.000
Diuretics	24	20	0.307
ACE inhibitors	25	25	1.000
Atrial fibrillation	6	10	0.372

NYHA = New York Heart Association class; EF = ejection fraction.

Only one patient in the control group had markedly elevated systolic pressure (63 mmHg) together with NYHA class III symptoms. This patient subsequently had a valve replacement seven months after the echocardiographic and NT-proBNP assessment.

The orifice area was markedly increased in 12 patients in the study group (Table 2). Group comparisons revealed that early diastolic filling ratios (E-mitral/E-annulus) was higher in the study group (*p* = 0.04). A similar pattern was observed with NT-proBNP level, which was elevated in both groups, but was markedly higher (*p* < 0.001) in the study group (Table 3).

**Table 2:** Baseline Quantification Of The Severity Of MR

*Variables*	*Controls (n = 27)*	*Study (n = 27)*	p-*value*
RF < 70%	6	2	
70–80%	6	3	0.192
> 80%	15	19	
RV > 60 ml	27	27	1.000
EOA < 0.35	3	1	
0.36–0.40	2	4	0.058
0.41–0.90	17	10	
> 0.90	5	12	
PAS < 30 mmHg	0	0	
30–39	10	6	0.192
40–59	16	18	
> 60 mmHg	1	4	
LA size < 40 mm	4	0	
40–49 mm	5	2	0.074
> 49 mm	20	25	
EF < 50%	0	1	
51–60%	1	5	0.123
> 60%	26	21	

RF = regurgitant fraction; RV = regurgitant volume; LA = left atrium; RV = right ventricle; PAS = pulmonary artery systolic pressure; EOA = effective orifice area; EF = ejection fraction.

**Table 3:** Pre-Operative Quantification Of LV Function

	*Controls*	*Study*	p-*value*
LA size (mm)	59.6 ± 13	76 ± 16	< 0.001
LA volume (ml)	177 ± 119	309 ± 183	0.003
EDV (ml)	177 ± 69	165 ± 48	0.472
ESV (ml)	58 ± 25	57 ± 25	0.898
EDD (mm)	67 ± 6	67 ± 9	0.06
ESD (mm)	38 ± 9	43 ± 8	0.043
EF	65 ± 10	67 ± 7	1.000
TDI (m/s)	0.09 ± 0.02	0.1 ± 0.07	0.377
TDI Em/Ea	14 ± 8	20 ± 8	0.004
NT-proBNP (pmol/l)	57 ± 88	262 ± 224	< 0.001

LA = left atrium; EDV = end-diastolic volume; ESV = end-systolic volume; EDD = end-diastolic dimension; ESD = end-systolic dimension; EF = ejection fraction, TDI = tissue Doppler imaging; TDI Em/Ea = diastolic filling ratio.

**Fig. 1:**
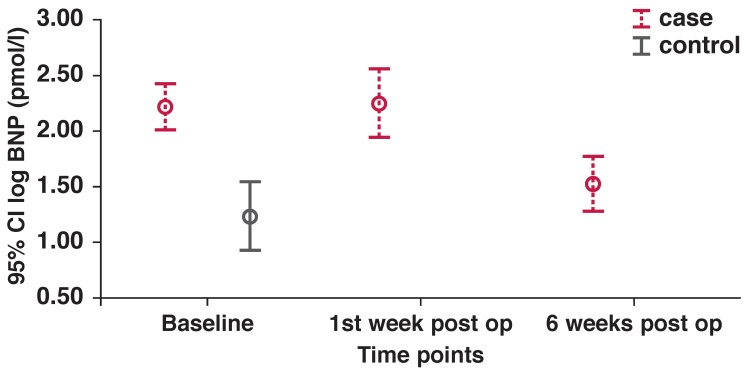
NT-pro BNP time-course changes.

The immediate post-operative period showed a rise in NT-proBNP levels from pre-operative levels (262 ± 224 pmol/l) to a mean of 395 pmol/l at one week, and subsiding thereafter to 94 pmol/l at six weeks (see Fig. 1). These changes were mirrored by a significant reduction in the left atrium (LA) size and volume, as well as in the LV chamber dimension to levels similar to the control population group (Table 4). It is noteworthy that in as much as there was a slight reduction in the end-diastolic volume (EDV) and end-systolic volume (ESV), these changes were not statistically significant. Although the TDI systolic wave indices were unchanged between the two time points, there was a significant increase noted in the early diastolic filling ratios, suggesting a further rise in the left ventricular filling pressures at six weeks.

**Table 4:** Post-Operative Evaluation At One And Six Weeks’ Follow Up

	*1 week*	*6 weeks*	*CI*	p-*value*
LA size (mm)	64 ± 16	58 ± 16	2.0; 11.2	0.009
LA volume (ml)	234 ± 152	182 ± 140	21.2; 82.9	0.003
EDV (ml)	139 ± 52	125 ± 55	–15.7; 43.8	0.326
ESV (ml)	86 ± 47	73 ± 47	–10.7; 37.5	0.250
EDD (mm)	59 ± 9	54 ± 8	2.5; 10.2	0.004
ESD (mm)	45.5 ± 13	38.7 ± 9	12.7; 1.0	0.03
EF	42 ± 13	51 ± 13	–17.9; –1.0	0.0032
TDI syst (m/s)	0.07 ± 0.016	0.07 ± 0.015	–0.01; -0.2	0.821
TDI Em/Ea	12 ± 4	15 ± 3	–6.7; –0.2	0.418
NT-proBNP (pmol/l)	395 ± 460	94 ± 161	87.5; 514.1	0.009

LA = left atrium; EDV = end-diastolic volume; ESV = end-systolic volume; EDD = end-diastolic dimension; ESD = end-systolic dimension; EF = ejection fraction, TDI = tissue Doppler imaging; TDI Em/Ea = diastolic filling ratio.

Four patients exhibited persistently elevated NT-proBNP levels at six weeks compared to the other study cases. The ejection fraction fell in three of these patients post-operatively, and was accompanied by little change in the end-systolic dimension (ESD) and NT-proBNP values compared to pre-operative levels. There was an increase in the diastolic filling ratios (Em/Ea) and a decrease in the TDI systolic wave. The fourth patient had a large drop in the NT-proBNP level, as well as an increase in the TDI systolic wave, in keeping with the preserved LV contractility. There was a small decrease in the ESD post-operatively (Fig. 1). These four were identified by NT-proBNP cut-off levels set by Januzzi’s rule-in criteria (450 pg/ml, ~53 pmol/l) for the diagnosis of heart failure in the PRIDE study^6^ at six weeks.

**Fig. 2:**
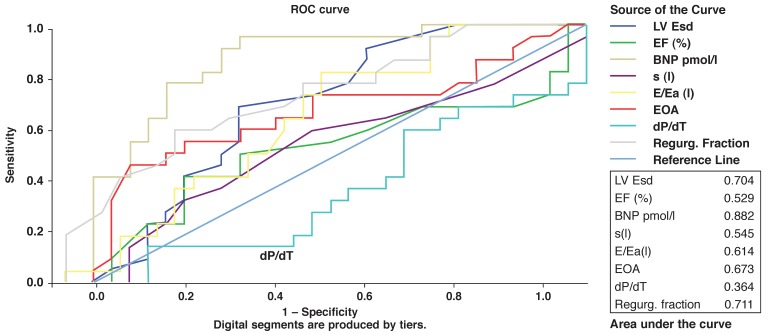
Receiver operating characteristics curve: surgical cases and controls.

A ROC curve was constructed for each variable to assess its discriminating capacity to distinguish between surgical cases and controls. The area under the curve was highest for NT-proBNP; this was the only parameter that approached 90% (Fig. 2). However, at established cut-off levels for normality, NT-proBNP (12 pmol/l = 125 pg/ml) yielded the highest sensitivity of 96% but had a low specificity of 45%. Using cut-off criteria established by Januzzi^6^ for the detection of heart failure (NT-proBNP > 53 pmol/l), the specificity for NT-proBNP improved to 74%, and the positive predictive value to 84%. Of interest, eight patients in the control group had BNP levels > 53 pmol/l, and of these, six underwent surgery within the ensuing six months.

**Fig. 3:**
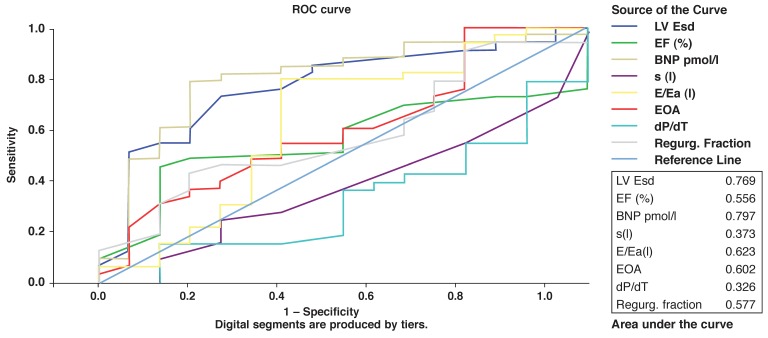
Receiver operating characteristics curve: NYHA all classes.

A second ROC curve (Fig. 3) was constructed using all the variables to separate NYHA classes I–II from III–IV. Once again, NT-proBNP emerged with the highest area under the curve, followed closely by the ESD. Bivariate analysis was performed to assess the ability of the different parameters to predict a favourable outcome, defined arbitrarily as NT-pro BNP level < 50 pmol/l. These were an effective orifice area (EOA) (*p* = 0.014), tissue Doppler S wave (*p* = 0.049) and left atrium (LA) size (*p* = 0.027), all pointing to the interrelationship between severe regurgitation, systolic function and NT-proBNP levels.

## Discussion

This is one of the first studies to use new modalities of measuring left ventricular function with TDI and NT-proBNP assays. NT-proBNP level has been shown to be a marker of left ventricular dysfunction and has been used to predict diastolic abnormalities in patients with normal systolic function,^7^ limiting the need for expensive cardiac imaging modalities.^8^,^9^ In this study, NT-proBNP level yielded the highest predictive value for discriminating between cases selected for surgery and controls followed up at the cardiology outpatients’ clinic (sensitivity of 96%). The ESD at the established cut-off point of 45 mm that defines the need for surgery had a higher specificity of 81%.

In a study similar to ours, Pillai *et al*.^10^ assessed pre-operative NT-proBNP levels in a group of 20 patients with rheumatic heart disease. They showed that elevated pre-operative NT-proBNP levels were an indicator of underlying myocardial dysfunction, which was not evident by routine two-dimensional echocardiography, and advocated pre-operative assessment of NT-proBNP levels to detect underlying myocardial dysfunction.

Two other studies have examined patients with varying degrees of MR, and showed that changes in ventricular function occur early in the disease process, even before they could be detected echocardiographically.^11^,^12^ The diastolic filling ratios were higher in the study group, indicating LV decompensation and a rise in the filling pressures. However, the diastolic ratios were also elevated in the control group, suggesting that LV decompensation with elevated LV filling pressure was already present in patients deemed by the clinician to be stable enough to be followed up at the clinic.

The six-week correlation between NT-proBNP levels and chamber dimensions suggests that in MR, changes in volume load may be paralleled by changes in the NT-proBNP level and that the fall in NT-proBNP was related to corrections in volume and removal of the diastolic run-off into the left atrium. These results are also in keeping with those found by the Mayo Clinic,^13^ which showed that the severity of mitral regurgitation, although univariately associated with NT-proBNP concentrations, was not an independent predictor of high NT-proBNP levels. They examined a group of 124 patients with varying degrees of organic mitral regurgitation and showed that NT-proBNP level was independently predictive of mortality/heart failure over a 4.4-year follow-up period.

Increased Em/Ea (> 12) ratios and elevated NT-proBNP (> 170 pg/ml) have been shown to be useful parameters to identify patients at increased risk of developing paroxysmal atrial fibrillation (AF) as well as to reflect early left ventricular dysfunction.^14^ We found that NT-proBNP and tissue Doppler levels in patients with AF were elevated, both in the study and in the control groups, indicating that symptomatology was not an early marker of ventricular decompensation and that our patients needed to be evaluated and referred for surgical intervention at an earlier stage in the course of their illness.

The challenge in evaluating mitral regurgitation is a determination of what really constitutes normal ventricular function in these patients. The limitations of using the ejection fraction in the timing of surgery become clear in subjects with apparently normal ejection fraction and minimal symptoms.

In a study of 84 asymptomatic patients who underwent surgical correction for MR, Agricola *et al*.^15^ demonstrated that TDI systolic indices could predict postoperative left ventricular function. In contrast, our study has shown that the TDI systolic wave cut-off point of 0.06 m/s, which has been previously used to rule out systolic dysfunction,^16^ had very low sensitivities in separating surgical cases from controls (see Fig. 2). Using a higher cut-off point of 0.085 m/s only marginally improved the specificity. It is possible that different cut-off points in conjunction with strain measurements^17^ may be more sensitive in determining impaired LV contractile function in MR, which can only be established in future studies with serial evaluations at different time intervals.

Left ventricular contractile dysfunction is present in many patients with severe MR despite a normal ejection fraction and returns to normal after corrective mitral valve surgery in most but not all patients.^18^ This was very apparent in our patients. In fact, all our patients experienced more than 10% decline in the EF immediately post surgery. This was improved at the six-week follow-up visit in all but three of our patients. Despite symptomatic improvement, postoperative left ventricular dysfunction (EF < 50%) has been shown to occur frequently, occurring in close to a third of the patients successfully operated on.^19^

In our study, 15 (15/27) of the patients referred for surgery had ejection fractions above 60% and ESD values below 45 mm, indicating that the reason for surgery in these patients was the presence of significant symptoms while on medical treatment. There is an inherent subjectivity in defining significant valve-related symptoms. Indeed, when the second ROC curve was constructed using NYHA class as the determinant, NT-proBNP again emerged as the strongest discriminator of advanced NYHA class.

The low specificity of 45% for NT-proBNP indicates a high number of false positive cases, i.e. patients selected by NT-proBNP level as requiring surgery when in fact they were still being followed up in the clinic on medical therapy. We believe that what is considered the high false-positive rate with NT-proBNP actually indicates that many of these control subjects with or without minimal symptoms actually required surgery, rendering them true positives. Waiting for more advanced symptoms or change in dimensions increases the risk of left ventricular dysfunction postoperatively. Two studies, both from the Mayo Clinic, have highlighted the poor outcomes in patients with severe MR who were managed conservatively rather than surgically.^20^,^21^

In another study, Pizzaro *et al*.^22^ reported that NT-proBNP level was a stronger prognostic marker than ESD or EOA and contributed independent prognostic information additional to other echo parameters. They showed that the BNP cut-off of 105 pg/ml (12.4 pmol/l for NT-proBNP) identified asymptomatic patients with severe MR who were at higher risk.

Most of the subjects in our control group had values above this level, indicating that all our patients were at risk while being followed up at the clinic. We attributed this to the subtle nature of symptoms, or particularly in our study, lack of awareness (on the part of patients or patients’ caregivers) of worsening symptoms and poor referral guidelines from peripheral healthcare centres. This low rate of intervention has also been reported in other centres as well.^23^ The Euro Heart Survey suggests that 31% of patients with severe valve disease and symptoms were not operated on.^24^

In our study, no parameter could discriminate pre-operatively between the four patients who exhibited persistently elevated NT-proBNP levels at six weeks and the rest of the sample. All four of these patients had atrial fibrillation pre-operatively, which persisted at six weeks and at six months. There was no indication of difficulties with myocardial preservation to suggest this as the cause for the decline in postoperative EF by almost 30% from normal pre-operative levels.

One of the limitations of this study was that it was performed only in patients with severe MR. The study needs to be repeated in different subgroups with varying degrees of MR in order to determine the time course of NT-proBNP levels in the different stages of MR as well as in patients with ischaemic MR. Finally, we need to determine the association of NT-pro BNP levels in a longer follow-up study with hard end-points such as heart failure and death.

## Conclusion

We have shown that decision making regarding the timing of surgery in this cohort of rheumatic heart disease patients was determined largely by advanced symptomatology, so that patients are referred to surgery late in the course of severe MR, with a risk of permanent LV decompensation. We propose that NT-proBNP level be an additional marker, particularly in less symptomatic patients, even if the EF is normal. In time, it may prove to be the composite marker for the assessment of LV decompensation. These findings support those of Detaint *et al*.^25^ in that the BNP level reflects the severe haemodymamic, ventricular and atrial consequences of MR.
